# Productivity Enhancement by Prediction of Liquid Steel Breakout during Continuous Casting Process in Manufacturing of Steel Slabs in Steel Plant Using Artificial Neural Network with Backpropagation Algorithms

**DOI:** 10.3390/ma15020670

**Published:** 2022-01-17

**Authors:** Md Obaidullah Ansari, Somnath Chattopadhyaya, Joyjeet Ghose, Shubham Sharma, Drazan Kozak, Changhe Li, Szymon Wojciechowski, Shashi Prakash Dwivedi, Huseyin Cagan Kilinc, Jolanta B. Królczyk, Dominik Walczak

**Affiliations:** 1 Department of Mechanical Engineering, Indian Institute of Technology, Dhanbad 826004, India; somuismu@gmail.com; 2 Department of Production and Industrial Engineering, Birla Institute of Technology Mesra, Ranchi 835215, India; joyjeet@bitmesra.ac.in; 3 Department of Mechanical Engineering, IK Gujral Punjab Technical University, Kapurthala 144603, India; 4 Department of Mechanical Engineering, University Centre for Research and Development, Chandigarh University, Punjab 140413, India; 5 Mechanical Engineering Faculty, University of Slavonski Brod, 35000 Slavonski Brod, Croatia; dkozak@unisb.hr; 6 School of Mechanical and Automotive Engineering, Qingdao University of Technology, Qingdao 266520, China; sy_lichanghe@163.com; 7 Faculty of Mechanical Engineering and Management, Poznan University of Technology, 60-965 Poznan, Poland; 8 GL Bajaj Institute of Technology & Management, Greater Noida, Gautam Buddha Nagar, Uttar Pradesh 201310, India; spdglb@gmail.com; 9 Department of Civil Engineering, Istanbul Esenyurt University, Istanbul 34510, Turkey; huseyincagankilinc@esenyurt.edu.tr; 10 Department of Manufacturing and Materials Engineering, Faculty of Mechanical Engineering, Opole University of Technology, Mikolajczyka 5, 45-271 Opole, Poland; d.walczak@po.edu.pl

**Keywords:** continuous casting, steel slab, mold breakout, artificial neural network, breakout prediction system

## Abstract

Breakout is one of the major accidents that often arise in the continuous casting shops of steel slabs in Bokaro Steel Plant, Jharkhand, India. Breakouts cause huge capital loss, reduced productivity, and create safety hazards. The existing system is not capable of predicting breakout accurately, as it considers only one process parameter, i.e., thermocouple temperature. The system also generates false alarms. Several other process parameters must also be considered to predict breakout accurately. This work has considered multiple process parameters (casting speed, mold level, thermocouple temperature, and taper/mold) and developed a breakout prediction system (BOPS) for continuous casting of steel slabs. The BOPS is modeled using an artificial neural network with a backpropagation algorithm, which further has been validated by using the Keras format and TensorFlow-based machine learning platforms. This work used the Adam optimizer and binary cross-entropy loss function to predict the liquid breakout in the caster and avoid operator intervention. The experimental results show that the developed model has 100% accuracy for generating an alarm during the actual breakout and thus, completely reduces the false alarm. Apart from the simulation-based validation findings, the investigators have also carried out the field application-based validation test results. This validation further unveiled that this breakout prediction method has a detection ratio of 100%, the frequency of false alarms is 0.113%, and a prediction accuracy ratio of 100%, which was found to be more effective than the existing system used in continuous casting of steel slab. Hence, this methodology enhanced the productivity and quality of the steel slabs and reduced substantial capital loss during the continuous casting of steel slabs. As a result, the presented hybrid algorithm of artificial neural network with backpropagation in breakout prediction does seem to be a more viable, efficient, and cost-effective method, which could also be utilized in the more advanced automated steel-manufacturing plants.

## 1. Introduction

**Figure 1:**
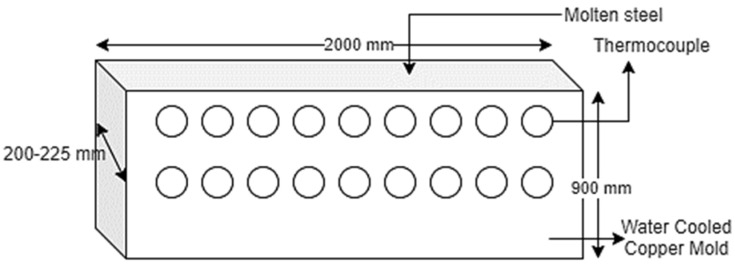
Copper mold for continuous casting at Bokaro Steel Plant.

Continuous casting of steel is a process in which liquid steel is continuously solidified into a strand of metal [1]. Depending on the dimensions of the strand, these semi-finished products are called slabs, blooms, or billets. Presently, most steel manufacturing industries worldwide use a continuous casting process, and more than 90% of the steel is produced by continuous casting [2,3]. Mold is the caster’s heart where the solidification process starts [4,5]. A slight difference in the mold may affect the productivity or quality of cast slabs [6,7]. At Bokaro Steel Plant (BSP), Bokaro, Jharkhand, India, straight mold is used and is made of copper, with a 900-mm length, a thickness of 200–225 mm, and a width of 2000 mm as shown in Figure 1.

**Figure 2:**
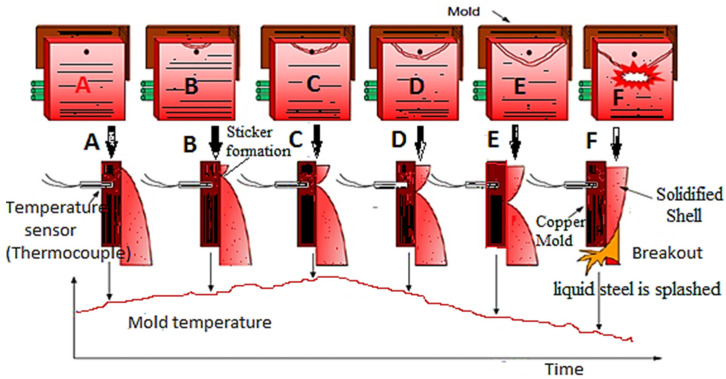
A schematic diagram of breakout.

Of all the problems present in slab caster, mold breakout of liquid steel is the most dangerous and hazardous problem. The liquid steel in the mold is slowly cooled and solidified to be drawn out from a lower portion of the mold as a strand. When the liquid steel is cooled within the mold, which is made of copper, a solidified portion is called a shell which is formed on the surface of the liquid steel. Sometimes semi-solidified slab sticks to the copper plate and forms cracks due to various factors. When a crack portion of the semi-solidified slab reaches the bottom of the mold, the liquid steel in the shell leaks out from the mold. This incident is called breakout, as shown in Figure 2. During a breakout, liquid steel is splashed, which leads to machinery damage, productivity, capital loss, safety hazards, and a temporary shutdown of continuous casting machines [8,9,10].

**Figure 3:**
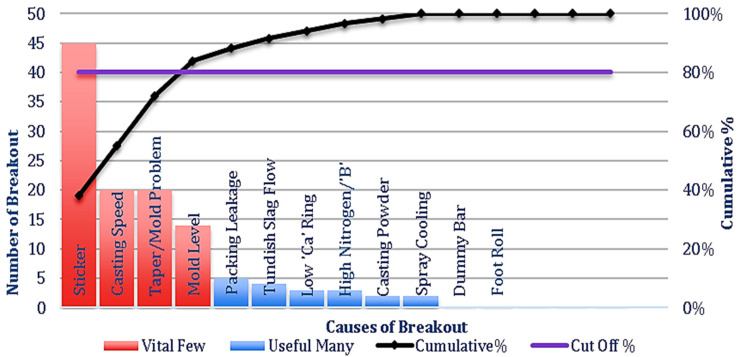
Pareto chart of causes of breakout.

The last five-year mold breakout data is collected from the operational logbook of BSP. From the data, it is clear that mold breakout can occur due to many reasons, but from the Pareto chart, it is clear that the first four causes of breakout cover 83.9% among all, i.e., sticker, taper/mold, casting speed, and mold level [11,12,13] as shown in Figure 3. More than 70–80% breakout occurs due to sticker breakout [14,15].

The total capital loss caused by the loss of liquid steel due to breakout per year is shown in Table 1. Approximately 31.15 million INR loss per year and an average delay due to liquid steel breakout are 94.4 h per year.

Mainly there are two types of breakout prediction systems based on thermocouple temperature or mold friction: Logical judgment method [16,17,18,19,20] and artificial intelligence method [21,22,23,24]. In BSP, a logical-based breakout prediction system is used to predict the breakout by using variation in multiple thermocouple temperatures. Logical judgment-based systems totally depend on thermocouple temperature, caster equipment, casting speed, mold friction, etc. [25,26] and generate false alarms or even fail to generate alarms before the breakout.

A literature survey shows that a diverse category of neural networks has been used to predict the breakout. Such as a data-driven multilayer perceptron-based artificial neural network model has been developed by using various casting process parameters [2]. Genetic algorithm-back propagation neural network has been used to construct a thermocouple temperature time series model [8]. A multilevel neural network based on the Takagi–Sugeno model [26], quantum wavelet neural network with multi-resolution based on the wavelet analysis theory, and the theory of quantum superposition [27], and a convolutional neural network by using sticker pattern in a mold heat-map [28], has also been used to predict the breakout. Support vector machine learning approach was also used to predict breakout [29]; it is based on the Vapnik–Chervonenkis theory and structural risk minimization principle. Nowadays, the breakout prediction system is based on K-mean clustering and dynamic time warping using a temperature change rate [30].

All the above-discussed methodology either used a thermocouple temperature or mold friction to predict the breakout. However, operational logbook data and Pareto chart show that mold breakout depends on various casting process parameters like casting speed, mold level, and mold/taper. 

For the first time, this work considered various parameters simultaneously; all of these parameters play an indispensable role in the production of steel slabs. Literature was not found in which all the parameters like casting speed [31,32,33], mold level [34,35,36], and taper/mold were used simultaneously to predict the breakout. The investigators of the prior studies have considered mainly one parameter in their study while simulating the casting result to predict the sticker-type breakout. In the current study, several process parameters like casting speed, mold level, thermocouple temperature, and taper/mold were considered during the manufacturing of steel slabs to predict different types of breakouts with the variation in the process parameters. For example, suppose the casting speed is too high during the continuous casting process of steel slabs. In that case, the liquid steel does not have enough time to solidify within the mold, thus causing another type of breakout. The authors have noticed a different kind of breakout that happens when the liquid steel level in the mold is less than 20% during processing, which ultimately causes mold-level breakout. Taper/mold is also an important parameter in continuous casting of steel slab [37,38,39,40,41]. Properly tapered mold compensates for shrinkage of the solidifying liquid steel to maintain good contact and heat transfer between the mold wall and semi-solidify steel slab. Improper mold taper provides an air gap between the mold wall and semi-solidify steel slab, which leads to breakout. Thus, properly tapering the mold before the casting process has been started is essential.

Thus, the authors have deeply analyzed all the breakouts as mentioned earlier during continuous casting of the steel slabs in this current work. The researchers of the previous studies have not used the hybrid optimization algorithm technique such as the integration of a hybrid neural network and artificial intelligence to simulate the casting result. Mostly, the researcher of the past studies has carried out their simulation results by using conventional approaches only. 

Therefore, a breakout prediction has been developed in this work by considering all 10 parameters, as shown in Table 2. This work has developed an artificial neural network with a backpropagation algorithm using Python to write the code and using the Keras format and TensorFlow as a backend. Keras is a deep learning application programming interface written in Python, running on top of the machine learning platform TensorFlow, focusing on enabling fast experimentation. The ANN-BP model is trained by using thermocouples temperature of a copper mold (T1, T2, T3… T8), casting speed (CS), and mold level (ML) as an input. The output of this model is either 0 (for no breakout) or 1 (for breakout). Whenever the breakout alarm is generated, casting speed will be reduced, and in case of a teeming interrupt, casting speed is reduced to creep’s speed.

## 2. Process Description

**Figure 4:**
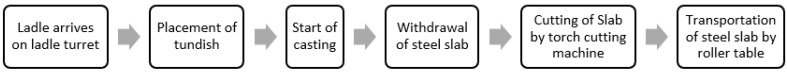
Process flow of continuous casting shop.

This study was performed at BSP, located at Bokaro, Jharkhand, India. Regarding materials and the processing method, the investigation study was carried on steel slab (materials) and correspondently the manufacturing method employed for the production of the steel slab in the production line of BSP was a continuous casting method. The structural layout of the specific shop floor where the manufacturing of steel slab was carried out using a continuous casting process is shown in Figure 4.

**Figure 5:**
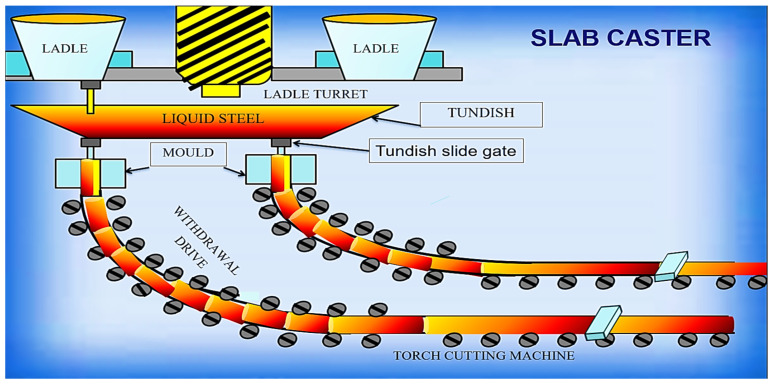
Schematic diagram continuous casting process.

**Figure 6:**
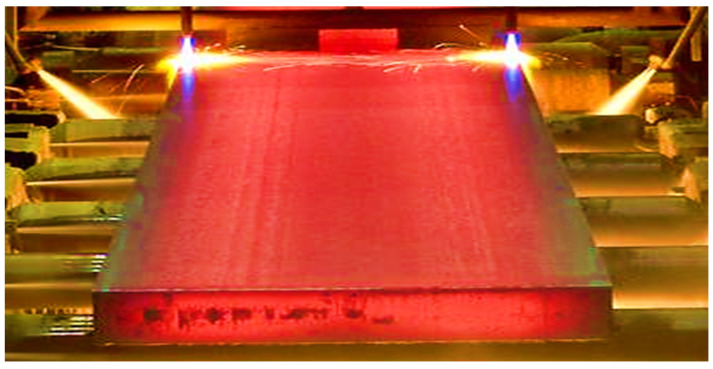
Desired product steel slab material.

The desired product produced on this shop floor is a steel slab, which is proceeded by a continuous casting method whose schematic diagram shown in Figure 5. The ladle, which carries liquid steel, is usually delivered by crane and positioned into a ladle turret, which subsequently rotates the ladle into the casting position. A slide gate in the bottom of the ladle is opened to allow the liquid steel to flow via a protective shroud into a tundish, a vessel that acts as a buffer between the ladle and mold. As the tundish fills, stopper rods are raised to allow the casting of steel into a water-cooled copper mold below the tundish. Solidification begins at the mold, and the steel is withdrawn as a metal strand. Throughout the entire casting process, the mold oscillates vertically in order to separate the semi-solidified steel from the copper mold. As the steel leaves the mold, it has only a thin solidified shell that needs further cooling to complete the solidification process. This is achieved in the so-called secondary cooling zone, in which a system of water sprays situated between the rolls are used to deliver a fine water mist onto the steel surface. Once it has completely solidified, the strand can be divided by a torch cutting machine and formed steel slab, as shown in Figure 6. These are either discharged to a storage area or to the hot rolling mill.

## 3. Methodology

### 3.1. Design Flow Chart of Breakout Prediction Model

**Figure 7:**
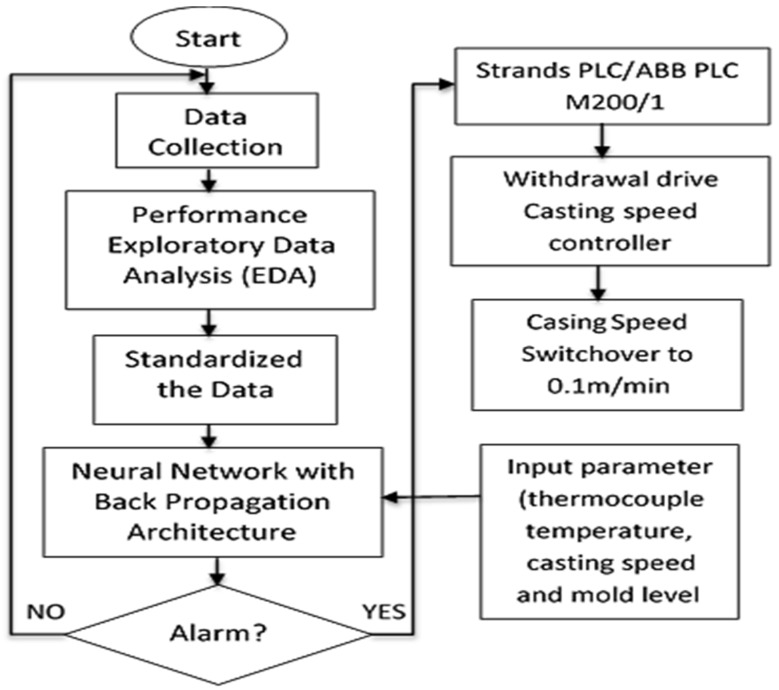
Flowchart diagram of the breakout prediction system.

This paper has developed an intelligence-based breakout prediction model, as shown in Figure 7. The model consists of three major parts: Exploratory data analysis (EDA), ANN-BP, and programmable logic controller (PLC) trigger. The first part is EDA, used better to understand data, their relationships, and pattern. The second part is ANN-BP, used to generate mold breakout alarm by using thermocouple temperature, casting speed, and mold level and sending a signal to strands PLC. The third part uses PLC to reduce casting speed whenever strands receive a breakout alarm signal. PLC automatically reduces the casting speed to 0.1 m/min through the withdrawal drive casting speed controller.

### 3.2. Exploratory Data Analysis (EDA)

EDA describes the data through statistical and visualization techniques better to understand the data, their relationships, and patterns. This is very important, primarily when data sets are used in deep learning, machine learning, neural network, etc. Here EDA is performed by using a correlation matrix and histogram.

#### 3.2.1. Correlation Matrix

**Figure 8:**
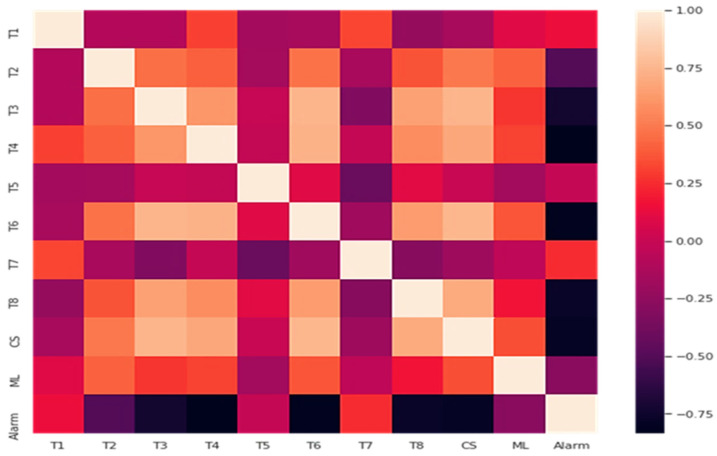
Correlation matrix.

The correlation between all the dataset variables is found using a correlation matrix, as shown in Figure 8. In the correlation matrix, the rows and columns represent the different variables of the dataset. Each cell of the matrix also contains the value of the correlation coefficient of the elements described by that particular row and column. If the value is near +1, there is a strong positive linear interdependency between the variables. In contrast, if it is nearer to −1, it indicates a strong negative linear correlation between the two parts. If the value is zero or near zero, there is no correlation between the two variables.

##### Calculation of the Correlation Coefficient

Calculation of correlation between two variables A and B. Standardized forms of *A* and *B* are *PA* and *PB*, respectively. *PA* and *PB* both have means equal to 0 and standard deviations (*S.D.*) equal to 1. Standardized schemes are obtained by using Equations (1) and (2) [35]:

(1) $$PA_{i}=\left[A_{i}-mean\left(A\right)\right]/S.D.\left(A\right)$$

(2) $$PB_{i}=\left[B_{i}-mean\left(B\right)\right]/S.D.\left(B\right).$$

The correlation coefficient is calculated as the mean product of the paired standardized scores (*PA_i_*, *PB_i_*) as expressed in Equation (3) [42]:

(3) $$R_{A,B}=Sum\;of\;\left[PA_{i}\times PB_{i}\right]/\left(n-1\right)$$

n=sample size.

From the above Figure 8, it is deduced that the relationship of alarm with T7 and T1 is positive and with CS, T8, T6, T4, T3, T2, and ML is negative and with T5 is almost equal to zero.

#### 3.2.2. Histogram

**Figure 9:**
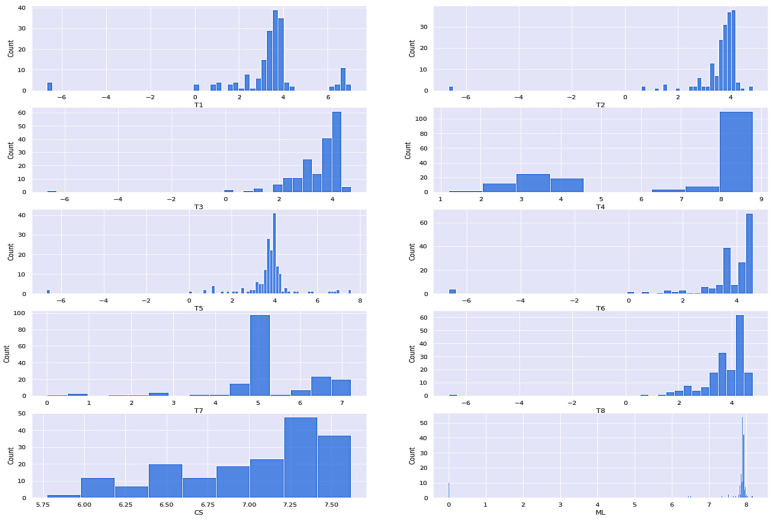
Histogram plots of the frequency distribution of all the variables (thermocouple temperatures (T1, T2 … T8), casting speed (CS), and mold level (ML).

The histogram represents the frequency distribution of all the variables. Now data analysis is done with the histogram and creates a frequency distribution of all the variables like thermocouple temperatures (T1, T2 … T8) and the other two were casting speed (CS) and the mold level (ML), as shown in Figure 9.

From the histogram plots (Figure 9), it can be inferred that the distribution does not follow a normal distribution, and the data is slightly skewed, but as the dataset is quite smaller, a small amount cannot also be afforded to be lost. To remove skewness, the most often used function is the Box–Cox power transform [43]. Box–Cox transformations hλ(x) are given by Equation (4):

(4) $$h_{a}\left(x\right)=f\left(x\right)=\{\begin{array}{c} \;\frac{\left\lfloor {\left(1+x\right)}^{\lambda }-1\right\rfloor }{\lambda },\;if\;\lambda \neq 0\;and\;x\geq 0\\ \;\log\left(1+x\right),\;\;if\;\lambda =0\;and\;x\geq 0\\ \frac{-\lfloor {\left(1-x\right)}^{2-\lambda }-1\rfloor }{\left(2-\lambda \right)},\;\;if\;\lambda \neq 2\;and\;x<0\\ -\log\left(1-x\right),\;\;if\;\lambda =2\;and\;x<0 \end{array}\;$$

Now skewness is removed from data with the help of Box–Cox transformations. After correlation and histogram, data is ready for training the neural network.

### 3.3. Data Pre-Processing

The following describes the process of making the raw data suitable for training purposes. A total of 786 sets of raw data were collected from the BSP. As there were variations within the data, it was normalized so that the total data was in the range of 0 to 1 before training the neural network.

#### 3.3.1. Train Test Split

Before standardizing data, the dataset was split into train and test sets, respectively, using the train-test-split function of the sklearn library. The train and test sets were standardized separately to obtain better accuracy.

#### 3.3.2. Standardizing the Data

Standardizing the data means transforming the data such that the mean and standard deviation of the distribution becomes 0 and 1, respectively. To achieve this, the mean value was subtracted from each value of the dataset and then divided by the standard deviation of the whole dataset. Since our dataset has multivariate data, this was done variables-wise, i.e., independently for each column and using the standard scaler from the sklearn.

**Figure 10:**
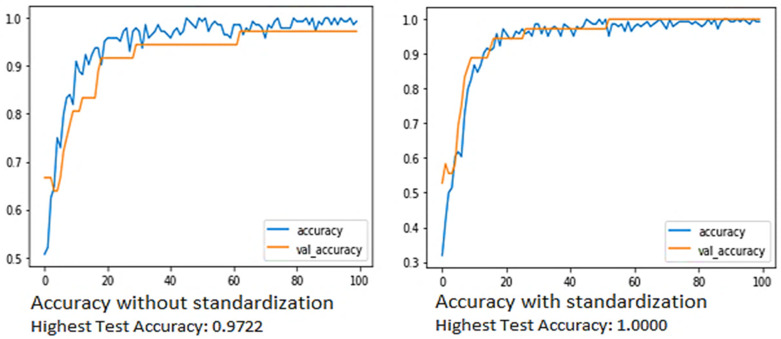
Accuracy curve without and with standardization.

The comparison between the accuracy curves of the model without and with standardization is given below. The accuracy curve shows that the model with a standardized data test accuracy of 1.0000 is found out to be higher compared to without, i.e., 0.9722 as shown in above Figure 10. After standardization, 505 sets of data were ready to train the neural network. A total 70% of data was used for neural network training, 15% of data was used for network validation, and 15% of data was used for model testing. Out of 505 samples of data, 100 sets of data samples belonged to breakout and pending 405 sets of data samples showed no breakout.

### 3.4. Neural Network Architecture

#### Hidden and Output Layers and Activation Function

**Figure 11:**
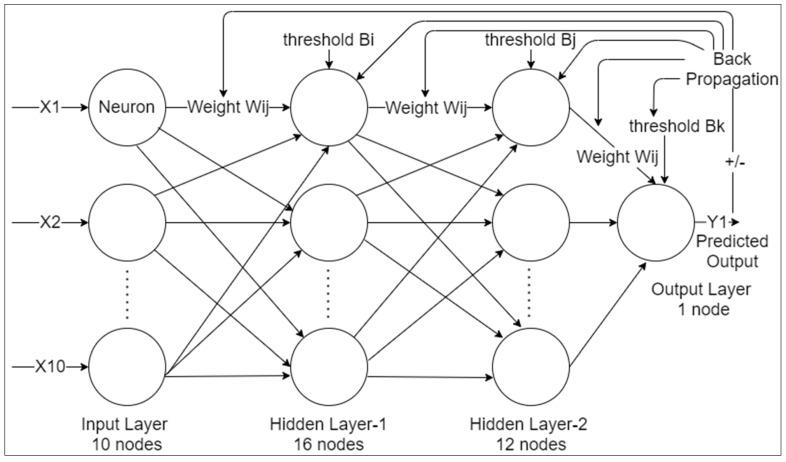
The architecture of the backpropagation neural network.

In artificial neural networks, the first layer is called the input layer; it consists of the 10 inputs, which are the different features of every dataset observation. The last layer is called the output layer, which contains one neuron as it is a binary classification task, and the layers present between the input and output layer are called the hidden layers. This neural network has two hidden layers, the first and second hidden layer have 16 and 12 neurons each, respectively, as shown in Figure 11. For the first and second hidden layer, the rectified linear unit (ReLu) activation function is used, and for the output layer, the sigmoid activation function is used. 

The sigmoid activation function is used majorly in the output layers of binary classification problems. It gives a value between 0 to 1, which is the probability prediction of the output, while the ReLu function is one of the most important frequently used activation functions in the hidden layers. It gives a better performance than the sigmoid activation function in the neural networks. Therefore, the sigmoid function is mainly used for the output layer and the ReLu function is used for the other two hidden layers for better performance. As the sigmoid function is used in the output layer, the predicted probability of mold breakout, which will be between 0 and 1, is calculated by the ANN. For the final values, these probabilities have to be converted to either 0 or 1 depending on the threshold value, which is 0.5 by default. 

The similar research has been documented in the previous study where the author calculated the predicted out by using neural network with the output layer compresses of one node [8,20,21].

### 3.5. Dropout and Batch-Normalization

The Dropout and Batch-Normalization technique has been used to improve the model performance after each of the two hidden layers. The dropout technique is used to prevent neural network models from overfitting. The dropout parameter is set to 0.3 for this model [44]. Batch-Normalization is one of the techniques which standardizes the input, which is fed to the layers for each of the mini-batch during the training of neural networks [45]. This helps stabilize the learning process and reduce the number of training epochs required during the training of the neural networks.

**Figure 12:**
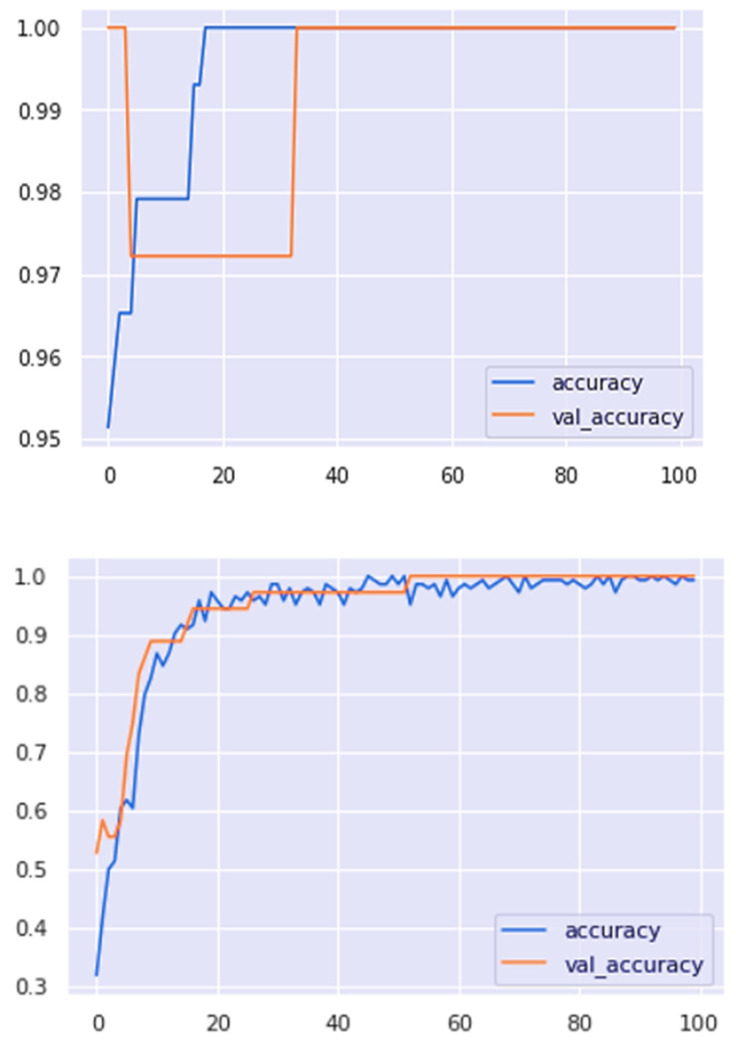
Accuracy curve of the model without and with dropout and batch normalization.

The comparison between the accuracy curves of the model without and with dropout and batch normalization is given below in Figure 12.

From the accuracy curve of the model without dropout and batch normalization, it can be deduced that the model is overfitted if dropout and batch normalization is not used.

### 3.6. Selection of Optimizer and Loss Function

Optimizers are the algorithms used to adjust the attributes of neural networks, such as weights and learning rates, to reduce the losses during the training process. In this model, the Adam optimizer is used as it efficiently combines the properties of the RMSProp and AdaGrad algorithms [46,47,48,49,50]. It is one of the most used optimizers in deep learning as its default parameters work well for most of the classification problems, and also, the sparse gradient for problems with noise can be easily handled by it. It is therefore efficient and comparatively faster. The binary cross-entropy loss function is used since our problem is a classification problem. In earlier research papers, MSE (mean squared error loss) was used, but it is used for regression tasks, not for classification tasks because the decision boundary in a classification task is significant (in comparison with regression). Setting the epochs to 100 and the batch size as 16 after implementing the above model, we gained a maximum of 100% accuracy in our training and testing sets. The binary cross-entropy loss function calculates the loss of a single output by using the following formula given by Equation (5).

(5) $$Loss=-\frac{1}{output\;size}\overset{output\;size}{\underset{i=1}{\sum }}y_{i}\log y_{i}+\left(1-y_{i}\right)$$

where $y_{i}$ is the *i*th scalar value in the model output, $y_{i}$ is the corresponding target value, and the output size is the number of scalar values in the model output.

## 4. Results and Discussions

### 4.1. Accuracy Curve

**Figure 13:**
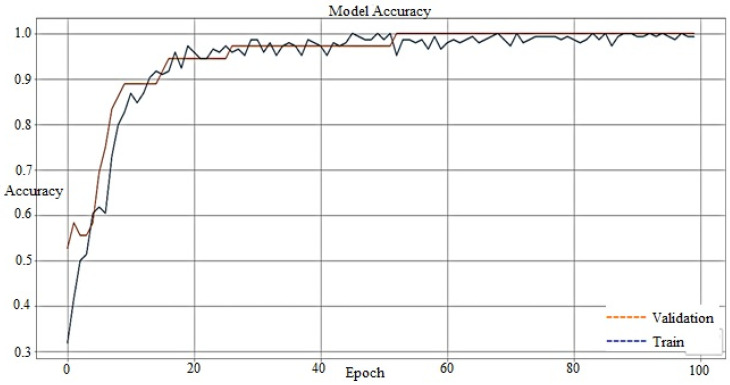
Accuracy curve of the model.

It has been seen that the accuracy of the model is increasing as we are increasing the number of epochs. Both the train and validation accuracy are rising to 100%, as shown in Figure 13.

### 4.2. Loss Curve

**Figure 14:**
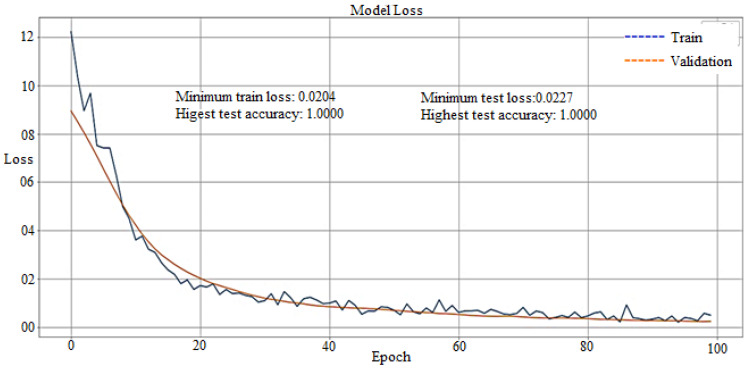
Loss curve of the model.

The loss curve of the model decreases with the number of epochs, as shown in Figure 14. Both train loss curve and validation loss curve of the model approaches towards zero with the number of epochs. 

The loss curve clearly shows that loss decreases with the number of epochs and the model having minimum train loss (0.0204) and minimum test loss (0.0227). The loss curve also indicates that both the train and test accuracy have become 100%, with the highest train accuracy (1.0000) and highest test accuracy (1.0000). The testing result shows that the model accurately predicts the breakout.

The developed system will automatically control the casting speed whenever the BOPS alarms or teeming interrupt commands are generated.

An artificial neural network with a backpropagation algorithm has been developed using Python to write the Keras format and TensorFlow as a backend. The model is trained by code using the thermocouples temperature of the copper mold, casting speed, mold/taper, and mold level. The test results in Figure 12 show that the accuracy curve of the model with dropout and batch normalization is more accurate than that of the model without dropout and batch normalization. The accuracy curve in Figure 13 shows that the accuracy increases with an increase in the number of epochs; then, the training loss and test accuracy become 100% accurate with the highest train accuracy (1.0000) and test accuracy (1.0000). At the same time, it is clear from the loss curve that model loss decreases with the number of epochs, and the model having a minimum train loss (0.0204) and minimum test loss (0.0227) is achieved in Figure 14. The experimental results show that the developed model has 100% accuracy for generating an alarm during the true breakout and thus, ultimately reduces the false alarm.

### 4.3. Representative Field Application-Based Validation Test

The developed BOPS is implemented for field trials from April 2021 to September 2021 to access the model’s performance and to check the accuracy. The data are collected for field tests from April 2021 to September 2021, with an average of 29.11 heat per day, i.e., 5299.02 casting heat in 6 months. There are 13 breakouts during this period, as shown in Table 3. Field application result shows that the developed BOPS can timely detect all 13 breakouts, and only 6 false alarms are generated. 

In other words, the detection ratio (detection ratio = number of true alarms/(number of missed alarms + number true alarms) for breakout, frequency of false alarms for presented model, and prediction accuracy ratio (prediction accuracy ratio = correct alarm times/(missed alarm times + true alarm times + false alarm times)) are 100%, 0.113%, and 100% respectively as shown in Table 4. The breakouts could be prevented because of timely alarm, thereby effectively reducing the casting speed and maintaining the mold level by opening and closing the tundish slide gate. Reducing the casting speed provides enough time for healing the sticker. The healing rate of the sticker has reached 100%. In the prevalent BOPS, there are 195 false alarms. The actual BOPS not only has a lot of false alarms, but that 13 breakouts were not detected, and thus the breakouts happened. A total loss of 31.15 million INR/year could be prevented in monetary terms. Therefore, the developed BOPS can be used effectively in the caster. The developed BOPS model can achieve better performance for breakout prediction and prevention and prevent huge financial loss.

Similar observations have been reported in prior studies, as shown in Table 5 for evaluating the breakout detection ratio (%), breakout prediction accuracy ratio (%), false alarm-time, and several false alarms [8,15,51]. Thus, our findings proved more reliable and substantial to prevent any breakout in steel industries as compare with the data of other researchers.

**Figure 15:**
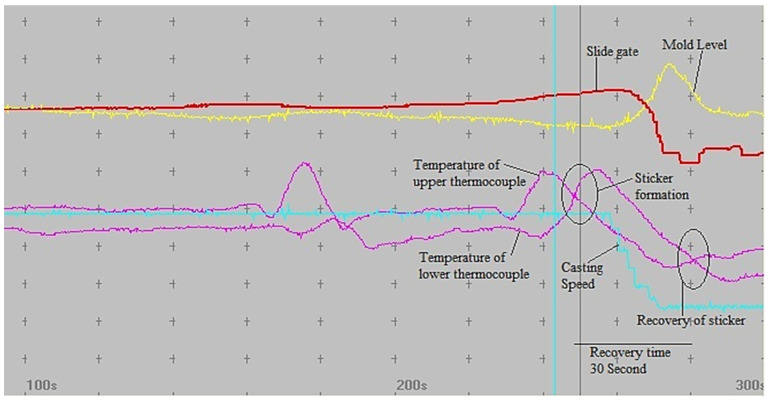
Representative breakout data of one field test (April 2021 to September 2021) depicting the recovery of a sticker-type breakout by reducing the casting speed.

A graphical illustration is shown in Figure 15, how the developed BOPS effectively prevents the breakout as an example of sample 7. The graphical illustration is shown in Figure 15 is of a single heat data. Here, the difference between the upper thermocouple temperature and lower thermocouple temperature exceeds the pre-defined value (40 °C), and hence, there is the formation of sticker-type breakout. Thereby, this condition releases a breakout alarm at 06:30:30. After the alarm, a breakout is prevented by reducing the casting speed, providing enough time to heal the sticker, and preventing the breakout. Thus, the sticker shell will be healed. Within 30 s, the sticker shell is completely healed. Therefore, there is a recovery of the sticker, which is shown in Figure 15.

## 5. Conclusions

In this work, an artificial neural network with backpropagation mold breakout prediction was developed using the Keras format and TensorFlow as a backend. The currently used breakout prediction system was developed using the thermocouple temperature or mold friction. This system uses the thermocouple temperature, mold level, and casting speed to predict the breakout. The accuracy curve, loss curve, and testing result show that this system successfully predicts all types of breakouts and even reduces to generate false alarm during casting compared to the existing BOPS system in the Bokaro Steel Plant. At the time of a breakout alarm, the casting speed will reduce to 0.8 m/min, and in case the teeming interrupt casting speed is reduced to creep’s speed, i.e., 0.1 m/min. The simulation results showed that the developed model had 100% accuracy for generating an alarm during the true breakout and thus, completely reduced the false alarm. Furthermore, field trials results showed that the developed BOPS had a detection ratio of 100%, the frequency of false alarm was 0.113%, and the prediction accuracy ratio was 100%, which is better than the existing system used in the continuous casting of steel slab at Bokaro Steel Plant. The developed BOPS could successfully prevent losses, with a monetary valuation of maybe 31.15 million INR/year. Therefore, it could be easily concluded that the developed BOPS is much more effective than the already installed BOPS and is highly recommended for deployment at the continuous casting shop at Bokara Steel Plant.

## 6. Future Outlook: Development of a Framework for Automatic Reduction of Casting Speed

**Figure 16:**
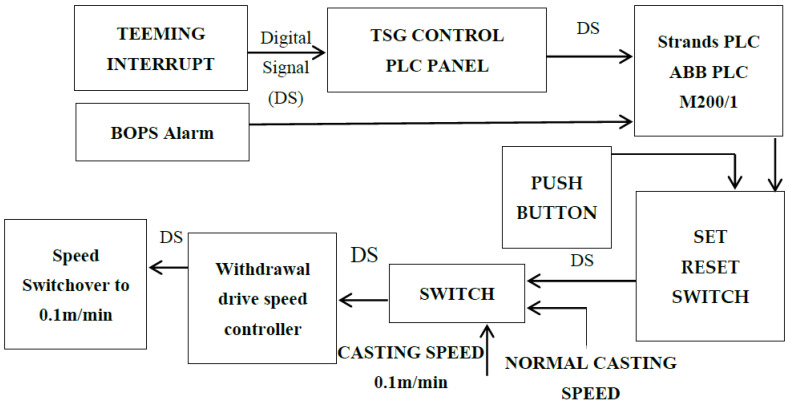
Proposed modem to reduce casting speed automatically.

The proposed model will automatically control the casting speed whenever there is a breakout alarm or teeming interrupt (when liquid steel level is increased within the mold, then the tundish slide gate is closed automatically and vice-versa. The tundish slide gate’s standard opening is a maximum of 78%, and a minimum is 48%. Whenever a tundish slide gate crossed this range, an interrupt command is generated by automatic mold level control (AMLC)) is generated. This model also controls the casting according to the grade of steel. When a teeming interrupt is generated, the casting speed range is reduced to creep’s rate, i.e., 0.1 m/min. In case of breakout alarm, the casting speed will reduce to 0.8 m/min, as shown in Figure 16.

In this proposed model, both the breakout alarm and teeming interrupt control the casting speed. Whenever teeming interrupt is generated simultaneously, TSG (tundish slide gate) control sends a command to the strands PLC (programmable logic controller). This strands PLC control the casting with the help of a withdrawal drive speed controller. In case of teeming interrupt casting speed, we will reduce it to 0.1 m/min. At the time of the breakout alarm, strands PLC well generates a signal to control the casting speed with the help of a withdrawal drive. In this case, a casting speed well reduces to 0.8 m/min.

## Figures and Tables

**Table 1:** Loss due to breakout.

Particular	Value
Number of breakouts	23.6 per year
After the breakout, an average of 3 to 4 h is required to restart the Caster. Average delay due to breakout = (4 × 23.6) = 94.4 h/year	
The average weight of liquid steel loss	3 ton per breakout
Total liquid steel loss	3 × 23.6 = 70.80 tons per year
Cost of one ton of liquid steel	44,000 INR
Loss of liquid steel per year	31.15 million INR/year

**Table 2:** Basic parameters of the model.

Input Parameters	Maximum Value	Minimum Value
Thermocouple’s temperature (T1, T2 … T8)	250 °C	50 °C
Casting speed	0.70 m/min	0.50 m/min
Speed setpoint	00 m/min	00 m/min
Mold level	Always greater than or equal to 20%	
Default upper thermocouple’s temperature always shows = 300 °C		
Default lower thermocouple’s temperature always shows = 0 °C		
Output parameter:	Output of this model is either ‘1’ for breakout and generate alarm or ‘0’ for no breakout and not generate any alarm	

**Table 3:** Breakout in continuous casting shop (CCS) from 1 April 2021 to 31 September 2021.

Breakout S.No.	Date and Time	Heat Number	Strand Number	Slab Size (mm)	Heat of Sequence	Ladle Number	Steel Grade	Casting Speed (m/min)	Mold Level (%)
		T1(°C)	T2(°C)	T3(°C)	T4(°C)	T5(°C)	T6(°C)	T7(°C)	T8(°C)
01	11 May 2021 06:30:30	53,969	02	1045	1st	14	CR2B	0.77	0
		186	8	146	9	10	76	−5	6
02	31 May 2021 04:10:02	54,335	02	1090	1st	21	GR-II	0.90	0
		188	12	132	3	−2	95	2	2
03	09 September 2021 03:17:56	57,263	04	1045	5th	13	GR-II	1.22	60
		13	130	20	14	−11	60	22	10
04	27 September 2021 22:50:31	57,803	4	1090	4th	9	CR	1.01	62
		15	6	15	10	21	46	4	−11
05	07 October 2021 06:17:59	58,132	1 & 2	1470/1320	8th	18	GR-II Patton	1.32	64
		178	32	178	14	−15	169	31	30
06	28 October 2021 23:17:38	58,898	2	1045	8th	23	CR2	1.09	54
		194	0	125	−12	−8	125	−3	-3
07	31 October 2021 06:30:30	58,974	4	1320	6th	17	GR-I	1.02	60
		4	10	−13	5	4	210	−12	5
08	09 September 2021 20:47:33	57,263	4	1045	5th	13	GR-II	0.78	0
		2	−1	1	0	−6	1	−13	7
09	27 September 2021 02:44:48	57,803	4	1090	4th	9	CR	0.50	1
		54	−14	55	−13	−17	63	−16	−18
10	07 October 2021 03:47:49	58,132	1 & 2	1470/1320	8th	18	GR-II Patton	0.38	0
		−38	−6	93	−5	−9	49	−4	−5
11	28 October 2021 12:49:60	58,898	2	1045	8th	23	CR2	1.08	39
		−1	−1	−5	−17	−15	208	−12	1
12	31 October 2021 20:55:03	58,974	4	1320	6th	17	GR-I	1.22	60
		19	133	21	10	−11	51	18	14
13	03 November 2021 13:51:14	59,060	3	1320	5th	25	GR-II	0.77	0
		189	8	146	9	10	79	−5	6

**Table 4:** Prediction results new and actual breakout prediction system (BOPS).

Parameters	New Model	Actual BOPS
Total number of heats	5299	5299
Total number of true breakout alarms	13	13
Total number of missed breakout alarms	00	13
Total numbers of false breakout alarms	06	21
Frequency of false alarms (%)	0.113	0.396
Breakout detection ratio (%)	100	50
Breakout prediction accuracy ratio (%)	100	27

**Table 5:** Compare the results obtained by the new model with the data of other researchers.

Authors	Breakout Detection Ratio	Breakout Prediction Accuracy Ratio	Frequency of False Alarm
New model	100%	100%	0.113
Liu, Yu, et al. [51]	98.73%	98.7%	0.126
He, Fei, et al. [15]	100%	78.26%	0.150
He, Fei, et al. [8]	100%	82.60%	0.1365

## Data Availability

The data presented in this study are available on request from the corresponding author.
